# Utilization of Radical Prostatectomy Versus Radiation Therapy for Gleason Grade Group 5 Prostate Cancer Before and After USPSTF Grade D Recommendation Against Prostate‐Specific Antigen Screening in 2012

**DOI:** 10.1002/cam4.70624

**Published:** 2025-02-05

**Authors:** H. Scott McGinnis, Taylor Corriher, James Janopaul‐Naylor, Subir Goyal, Yuan Liu, Zelin Wang, Sagar A. Patel

**Affiliations:** ^1^ Department of Radiation Oncology Winship Cancer Institute, Emory University Atlanta Georgia USA; ^2^ Department of Radiation Oncology Memorial Sloan Kettering Cancer Center New York New York USA; ^3^ Department of Epidemiology and Biostatistics The University of Arizona Tucson Arizona USA

**Keywords:** adjuvant radiotherapy, prostate cancer, prostatectomy, salvage radiotherapy

## Abstract

**Objectives:**

The 2012 United States Preventive Services Task Force (USPSTF) Grade D recommendation against prostate‐specific antigen (PSA) screening has resulted in a shift to higher‐stage prostate cancer (PC) at diagnosis. We evaluate the utilization of radical prostatectomy (RP) versus radiation therapy (RT) in the US for Gleason grade group 5 (GG5) prostate cancer before and after 2012.

**Methods:**

We identified 34,011 men with localized GG5 PC undergoing primary therapy with (1) RP or (2) RT + androgen deprivation therapy (ADT) between 2004 and 2017 from the National Cancer Database. The chi‐square test was used to compare the relative use of RP and RT before versus after 2012. Annual use of RP versus RT from 2004 to 2017 was compared using Cochran‐Armitage test for trend. We modeled the effect of treatment year on the use of RP using multivariable logistic regression.

**Results:**

Across all centers, the use of RP increased from 31% to 41% (*p* for trend < 0.001). 2012 was associated with significant inflection for increase in RP use in all centers. There was an increased odds of receiving RP after 2012 (adjusted OR 1.34, 95% CI 1.28–1.40, *p* < 0.001).

**Conclusions:**

Utilization of RP for GG5 PC has significantly increased in the United States over the past decade. It remains unknown if outcomes may be compromised in this group of high‐risk men, many of whom require post‐prostatectomy RT and/or ADT. Prospective comparison of RP versus RT + ADT for GG5 PC are needed to determine optimal treatment for these patients.

## 1 Introduction

Prostate cancer (PC) is the most common solid organ malignancy in men in the United States, with an estimated 288,300 new cases and 34,700 deaths from prostate cancer in 2023 alone [1]. The incidence of prostate cancer has increased by 3% per year from 2014 to 2019, which may be driven by increased incidence of high‐risk and advanced stage disease. The rise in high‐risk disease is largely attributed to modified prostate‐specific antigen (PSA) screening patterns, after the United States Preventive Services Task Force (USPSTF) issued a grade D recommendation against routine PSA screening for all men in 2012 [2].

Now, up to 20% of new prostate cancer diagnoses are considered high‐risk, characterized by initial PSA > 20 ng/mL, biopsy Gleason grade group (GG) 4 or 5, and/or clinical stage T3‐4 [3, 4]. Definitive treatment options include radiation (RT) plus androgen deprivation therapy (ADT) or radical prostatectomy (RP) [3]. For men pursuing RP, a substantial proportion will require post‐operative radiation therapy (RT) and/or ADT [5, 6]. The optimal treatment remains controversial with sparse prospective data [7]. Two recent studies focusing exclusively on patients with Gleason grade group 5 (GG5), high‐risk prostate cancer underscored the importance of multimodal therapy, including the use of adjuvant RT and/or ADT for men pursuing upfront RP [8, 9].

With the rise of high‐risk prostate cancer in the United States, its impact on first‐line therapy utilization, specifically RP versus primary RT with curative intent, remains unknown. Understanding evolving practice patterns for patients with high‐risk disease may support the need for future comparative prospective trials or guide professional guidelines, such as highlighting the importance of adjuvant therapy consideration in these men at the highest risk of recurrence after RP. Herein, we evaluate the utilization of RP versus RT in the United States for GG5, high‐risk prostate cancer before and after 2012 to assess treatment trends specifically associated with the evolving epidemiology of prostate cancer.

## 2 Methods

### 2.1 Data Source and Study Population

The National Cancer Data Base (NCDB), a nationwide hospital‐based cancer registry jointly sponsored by the American College of Surgeons and the American Cancer Society, collects data from more than 1500 Commission on Cancer‐accredited hospitals and captures approximately 70% of incident cancer cases in the US annually [10]. Data accuracy and quality are continually validated via data quality review, site surveys, and internal monitoring [11]. Because the study used deidentified data from the NCDB, the requirement for formal institutional review and the need for informed consent were waived, consistent with the policies of Emory University School of Medicine, Atlanta, GA.

Using the NCDB, we identified men with localized, very‐high‐risk, GG5 PC undergoing definitive, primary treatment with (1) RP or (2) RT plus ADT between 2004 and 2017. All patients met criteria for very‐high‐risk disease, defined in accordance with National Comprehensive Cancer Network guidelines as clinical stage T1‐4 and PSA > 20, or clinical stage T3‐4 and any PSA [3]. Those with node‐positive or metastatic disease were excluded. Any patient receiving simple prostatectomy, chemotherapy, immunotherapy, or palliative‐intent therapy prior to definitive, primary therapy was excluded as well. Patients who received additional therapy at time of relapse or development of metastatic disease were not excluded. Time to initiation of treatment data was also collected. This was defined as the number of days from diagnosis to surgery (RP) and number of days from initial diagnosis to initiation of RT (RT + ADT).

### 2.2 Statistical Analysis

Descriptive statistics were used to summarize baseline characteristics. Covariates included year at diagnosis, age at diagnosis, clinical T‐stage, biopsy Gleason score (Gleason 9 vs. 10), race, Charlson–Deyo Score, insurance provider, patient distance from treatment facility, facility type (academic vs. non‐academic), facility location (geographic region), urban versus rural setting, residential median income, and residential educational level. ANOVA and chi‐square test were used to compare clinical and demographic characteristics between those treated with RP versus RT + ADT.

The primary endpoint was the utilization of RP relative to RT before and after 2012, corresponding to the year of USPSTF Grade D recommendation against PSA screening. Chi‐square was used to compare the relative use of RP and RT before versus after 2012. Annual use of RP versus RT from 2004 to 2017 was compared in academic and non‐academic centers using Cochran–Armitage test for trend. Finally, we modeled the effect of treatment period (i.e., 2004–2011 versus 2012–2017) on use of RP using multivariable logistic regression. α tests were two‐sided with a 0.05 level of significance. Analyzes used SAS, version 9.4 (SAS Institute Inc) and SAS macros developed by the Biostatistics Shared Resource at Winship Cancer Institute in Atlanta, GA [12].

## 3 Results

**FIGURE 1:**
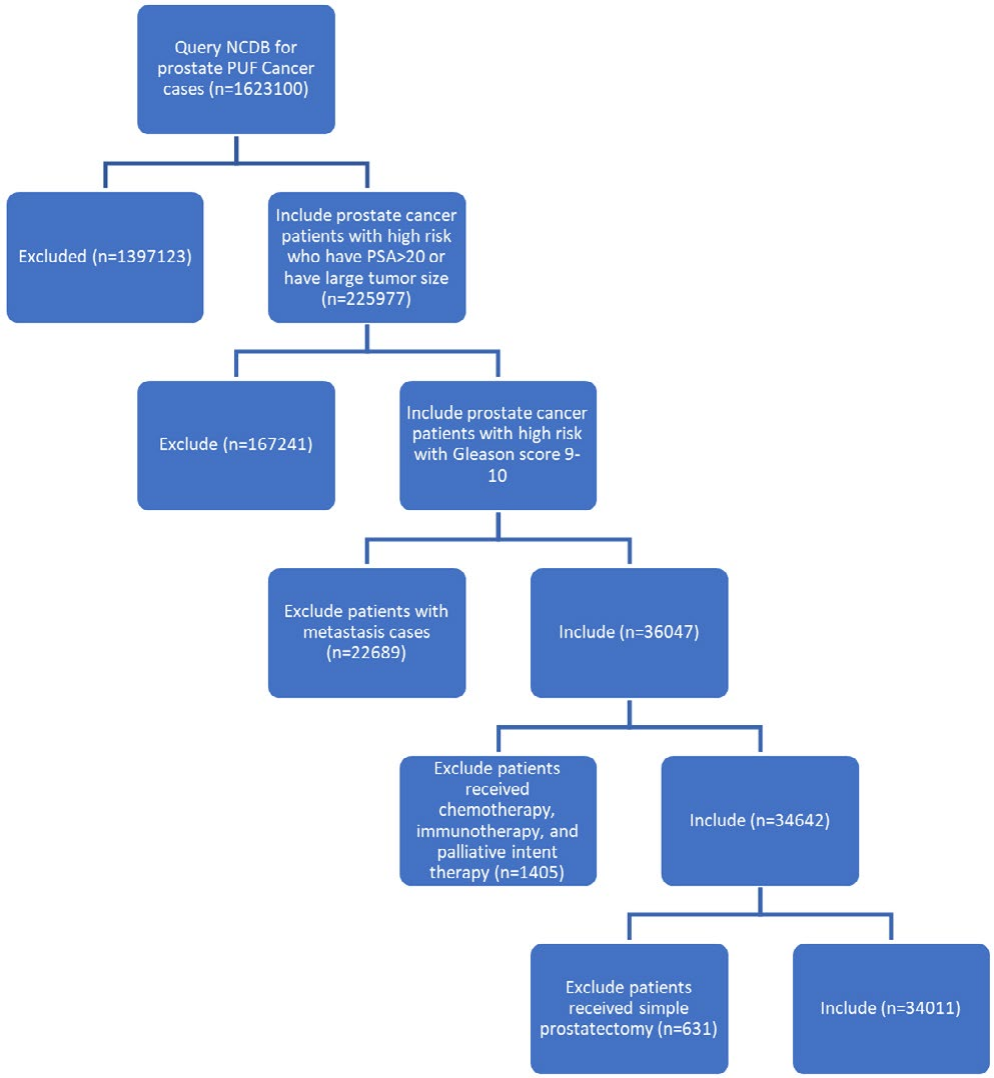
Consort diagram. Across all facility types in the US, use of RP increased from 31% in 2004 to 41% in 2017 (*p* for trend < 0.001) (Figure 2). Trends were similar in academic centers (32%–44%, *p* < 0.001) and non‐academic centers (30%–39%, *p* < 0.001) (Figures 3 and 4). Between 2004 and 2011, 36.5% (*n* = 5483) underwent RP; between 2012 and 2017, 42.3% (*n* = 8034) underwent RP (*p* = 0.02) (Figure 5).

Figure 1 shows the schema used to identify the eligible study cohort of 34,011 patients with very‐high‐risk, GG5 prostate cancer. Among this cohort, 13,517 (39.7%) patients were treated with upfront RP and 20,494 (60.3%) were treated with primary RT with curative intent +ADT. Compared with men treated with primary RT with curative intent, those treated with RP were more frequently ≤ 62 years old, White race, less comorbid (i.e., Charlson‐Deyo score 0–1) and covered by private/commercial insurance. All clinical and demographic covariates between treatment cohorts are shown in Table 1.

**FIGURE 2:**
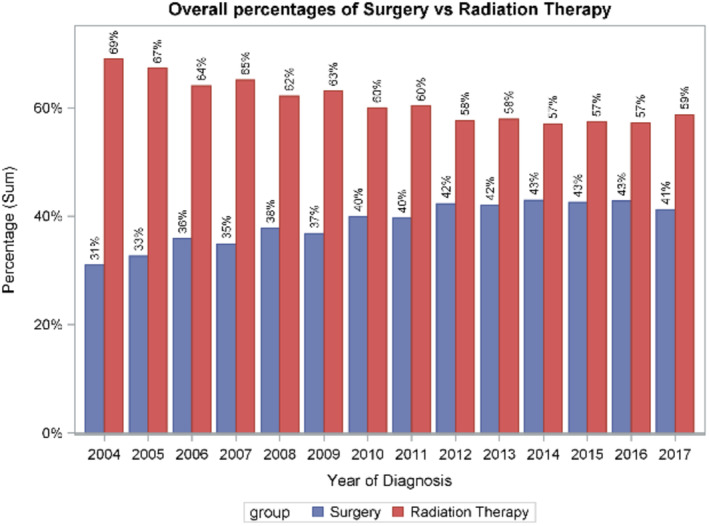
Overall percentages of surgery versus radiation therapy (RT), illustrating 31% of patients undergoing surgery in 2004 compared to 41% of patients in 2017.

**FIGURE 3:**
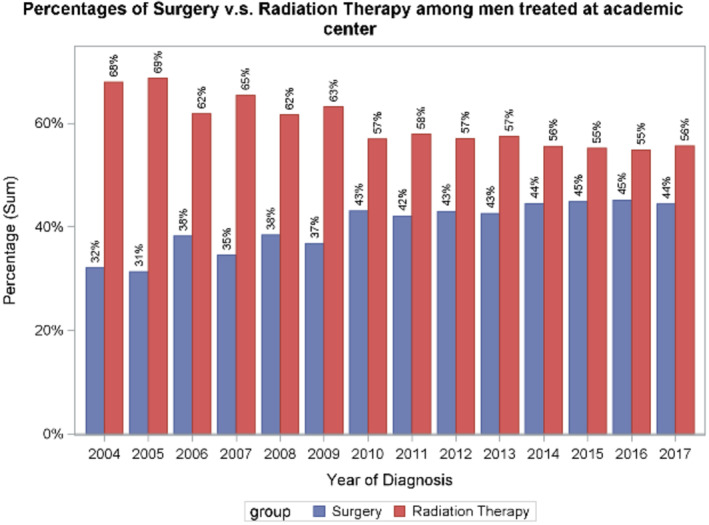
Overall percentage of surgery versus radiation therapy (RT) at academic centers, demonstrating the increase in surgery from 32% in 2004 to 44% in 2017.

**TABLE 1:** Descriptive statistics.

		Group		
Covariate	Total *N* = 34,011 (100%)	Surgery *N* = 13,517 (39.8%)	Radiation therapy *N* = 20,494 (60.2%)	*P‐value* ^*^
Age at diagnosis|*N* (%)				
Aged under 62	8338 (24.6)	4473 (33)	3865 (18.8)	< 0.001
Aged between 62 and 68	9083 (26.8)	4177 (31)	4906 (24)	
Aged between 68 and 76	8762 (25.8)	2830 (21)	5932 (29)	
Aged over 76	7828 (23)	2037 (15)	5791 (28.2)	
Race|*N* (%)				
White	24,966 (73.4)	10,403 (77)	14,563 (71)	< 0.001
Black	5550 (16.4)	1751 (13)	3799 (18.6)	
Asian‐Indians‐Pacific Islander	1064 (3.2)	442 (3.2)	622 (3)	
Hispanic	1858 (5.4)	715 (5.2)	1143 (5.6)	
Other/Unknown	573 (1.6)	206 (1.6)	367 (1.8)	
Charlson‐Deyo Score|*N* (%)				
0	27,175 (80)	10,432 (77.2)	16,743 (81.6)	< 0.001
1	4983 (14.6)	2331 (17.2)	2652 (13)	
≥ 2	1853 (5.4)	754 (5.6)	1099 (5.4)	
Primary payor|*N* (Col %)				
None/Unknown	2215 (6.6)	727 (5.4)	1488 (7.2)	< 0.001
Private	11,493 (33.8)	5798 (42.8)	5695 (27.8)	
Medicaid	1504 (4.4)	500 (3.6)	1004 (4.8)	
Medicare	18,799 (55.2)	6492 (48)	12,307 (60)	
Great circle distance|*N* (%)				
< 25 miles	23,316 (68.6)	8550 (63.2)	14,766 (72)	< 0.001
25–50 miles	3985 (11.8)	1682 (12.4)	2303 (11.2)	
≥ 50 miles	3754 (11)	2001 (14.8)	1753 (8.6)	
Unknown	2956 (8.6)	1284 (9.4)	1672 (8.2)	
Great circle distance				
Total *N*	34,011	13,517	20,494	< 0.001^**^
Mean (SD)	8724.8 (28160.8)	9540.4 (29308.2)	8186.8 (27365.2)	
Median (Q1–Q3)	12 (4.8–35)	14.4 (5.6–48)	10.8 (4.4–29)	
Min–Max	0–99,999	0–99,999	0–99,999	
PSA|*N* (%)				
< 10	4981 (14.6)	2250 (16.6)	2731 (13.4)	< 0.001
10–20	3169 (9.4)	1069 (8)	2100 (10.2)	
> 20	24,822 (73)	9499 (70.2)	15,323 (74.8)	
Unknown	1039 (3)	699 (5.2)	340 (1.6)	
PSA				
Total *N*	34,011	13,517	20,494	< 0.001^**^
Mean (SD)	344 (1714.2)	552 (2206.4)	206.6 (1272.2)	
Median (Q1–Q3)	31.8 (20.4–60.2)	30.4 (20.2–57)	32.4 (20.6–62.6)	
Min–Max	0.2–9999	0.2–9999	0.2–9999	
Facility type|*N* (%)				
Non‐academic	20,730 (61)	7995 (59.2)	12,735 (62.2)	< 0.001
Academic	13,268 (39)	5513 (40.8)	7755 (37.8)	
Facility location|*N* (%)				
Northeast	6981 (20.6)	2473 (18.4)	4508 (22)	< 0.001
South	11,353 (33.4)	4580 (34)	6773 (33)	
Midwest	9443 (27.8)	3839 (28.4)	5604 (27.4)	
West	6221 (18.2)	2616 (19.4)	3605 (17.6)	
Urban/rural 2013|*N* (%)				
Metropolitan	27,167 (79.8)	10,682 (79)	16,485 (80.4)	< 0.001
Urban	4911 (14.4)	1956 (14.4)	2955 (14.4)	
Rural	721 (2.2)	271 (2)	450 (2.2)	
Unknown	1212 (3.6)	608 (4.4)	604 (3)	
Census median income quartiles 2008–2012|*N* (%)				
< $38,000	5833 (17.2)	2071 (15.4)	3762 (18.4)	< 0.001
$38,000–$47,999	7209 (21.2)	2786 (20.6)	4423 (21.6)	
$48,000–$62,999	8265 (24.4)	3286 (24.4)	4979 (24.2)	
≥ $63,000	9706 (28.6)	4078 (30.2)	5628 (27.4)	
Unknown	2998 (8.8)	1296 (9.6)	1702 (8.4)	
Percentage without high school degree 2008–2012|*N* (%)				
≥ 21%	5655 (18.2)	2034 (16.6)	3621 (19.2)	< 0.001
13.0%–20.9%	7792 (25.2)	2941 (24)	4851 (25.8)	
7.0%–12.9%	10,024 (32.4)	4086 (33.4)	5938 (31.6)	
< 7.0%	7560 (24.4)	3164 (25.8)	4396 (23.4)	
Clinical stage|*N* (%)				
T1–T2	18,915 (55.6)	7315 (54.2)	11,600 (56.6)	< 0.001
T3–T4	10,745 (31.6)	4364 (32.2)	6381 (31.2)	
Unknown	4351 (12.8)	1838 (13.6)	2513 (12.2)	
Gleason Score|*N* (%)				
1	3811 (11.2)	1294 (9.6)	2517 (12.2)	< 0.001
9	30,200 (88.8)	12,223 (90.4)	17,977 (87.8)	
Year of diagnosis|*N* (%)				
2004–2007	6625 (19.4)	2240 (16.6)	4385 (21.4)	< 0.001
2008–2011	8386 (24.6)	3243 (24)	5143 (25)	
2012–2015	7863 (23.2)	3339 (24.8)	4524 (22)	
2015–2017	11,137 (32.8)	4695 (34.8)	6442 (31.4)	
Age at diagnosis				
Total *N*	34,011	13,517	20,494	< 0.001^**^
Mean (Std Dev)	68.6 (10)	66.2 (10)	70.4 (9.6)	
Median (Q1–Q3)	68 (62–76)	65 (59–72)	70 (63–77)	
Min–Max	33–90	33–90	33–90	
Time to treatment initiation (days)				
Total *N*	31,028	13,203	17,825	< 0.001^**^
Mean (Std Dev)	52.2 (61.6)	62.4 (69)	44.6 (54.2)	
Median (Q1–Q3)	40 (17–69)	53 (25–82)	31 (15–56)	
Min–Max	0–2587	0–2587	0–1751	

* The *p*‐value is calculated by either parametric (ANOVA, Cochran‐Armitage Trend) or non‐parametric (Kruskal–Wallis, Exact Cochran‐Armitage Tend) test, where appropriate, based on the normality test of data distribution and the sample size.

** A non‐parametric test (Kruskal–Wallis or Exact Cochran‐Armitage Tend test) is applied.

On multivariable analysis, there was a significantly increased odds of receiving RP between 2012 and 2017 compared to 2004–2011 (adjusted OR 1.34, 95% CI 1.28–1.40, *p* < 0.001). Full multivariable model is shown in Table 2. White race, age < 62, Charlson‐Deyo comorbidity score 0, private insurance, PSA < 10, clinical stage T1‐2, Gleason score 9, and high residential income were significantly associated with receipt of RP over primary RT with curative intent +ADT.

**FIGURE 4:**
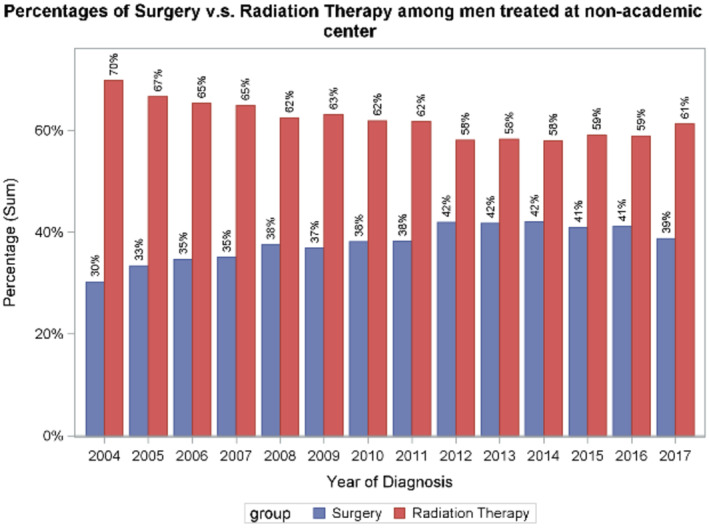
Overall percentage of surgery versus radiation therapy (RT) at non‐academic centers, also showing an increase of surgery from 30% to 39% from 2004 to 2017.

**TABLE 2:** Multivariable logistic regression modeling the likelihood of upfront radical prostatectomy.

			Group = Surgery		
Covariate^b^	Level	*N* ^a^	Odds ratio (95% CI)	OR P‐value	Overall P‐value
Year Group	2012–2017	16,694	1.32 (1.26–1.39)	< 0.001	< 0.001
	2004–2011	14,326	—	—	
Race	NH‐Black	5064	0.61 (0.56–0.65)	< 0.001	< 0.001
	Asian‐Indians‐Pacific Islander	964	1.05 (0.91–1.21)	0.514	
	Hispanic	1708	0.91 (0.82–1.02)	0.115	
	Other/Unknown	522	0.74 (0.61–0.89)	0.002	
	NH‐White	22,762	—	—	
Age	Aged between 62 and 68	8211	0.69 (0.65–0.74)	< 0.001	< 0.001
	Aged between 68 and 76	7985	0.38 (0.35–0.41)	< 0.001	
	Aged over 76	7282	0.28 (0.26–0.31)	< 0.001	
	Aged under 62	7542	—	—	
Charlson–Deyo Score	1	4578	1.54 (1.44–1.65)	< 0.001	< 0.001
	≥ 2	1692	1.33 (1.20–1.48)	< 0.001	
	0	24,750	—	—	
Primary payor	None/Unknown	2045	0.61 (0.55–0.68)	< 0.001	< 0.001
	Medicaid	1399	0.61 (0.54–0.69)	< 0.001	
	Medicare	17,152	0.91 (0.85–0.97)	0.005	
	Private	10,424	—	—	
Great circle distance	25–50 miles	3980	1.26 (1.17–1.36)	< 0.001	< 0.001
	≥ 50 miles	3743	1.84 (1.69–2.01)	< 0.001	
	Unknown	7	0.93 (0.17–5.16)	0.936	
	< 25 miles	23,290	—	—	
PSA	10–20	2866	0.65 (0.59–0.72)	< 0.001	< 0.001
	> 20	22,678	0.82 (0.75–0.88)	< 0.001	
	Unknown	981	3.47 (2.97–4.04)	< 0.001	
	< 10	4495	—	—	
Facility type	Non‐academic	19,098	1.04 (0.98–1.10)	0.164	0.164
	Academic	11,922	—	—	
Facility location	South	10,310	1.31 (1.22–1.41)	< 0.001	< 0.001
	Midwest	8570	1.29 (1.20–1.39)	< 0.001	
	West	5674	1.29 (1.19–1.39)	< 0.001	
	Northeast	6466	—	—	
Urban/rural 2013	Metropolitan	24,904	1.19 (1.10–1.29)	< 0.001	< 0.001
	Rural	653	0.86 (0.72–1.03)	0.097	
	Unknown	972	1.30 (1.12–1.51)	< 0.001	
	Urban	4491	—	—	
Census median income quartiles 2008–2012	<$38,000	5831	0.86 (0.78–0.95)	0.003	0.013
	$38,000–$47,999	7208	0.89 (0.82–0.97)	0.008	
	$48,000–$62,999	8260	0.92 (0.86–0.99)	0.024	
	Unknown	18	0.37 (0.11–1.19)	0.095	
	≥ $63,000	9703	—	—	
Percent No High School Degree 2008–2012	13.0%–20.9%	7792	1.00 (0.92–1.08)	0.925	0.469
	7.0%–12.9%	10,020	1.05 (0.96–1.15)	0.293	
	< 7.0%	7557	1.05 (0.95–1.17)	0.340	
	≥ 21%	5651	—	—	
Clinical stage	T3–T4	9793	0.93 (0.87–1.00)	0.038	< 0.001
	Unknown	4053	1.18 (1.09–1.27)	< 0.001	
	T1–T2	17,174	—	—	
Gleason score	10	3525	0.81 (0.75–0.88)	< 0.001	< 0.001
	9	27,495	—	—	

^a^ Number of observations in the original data set = 34,011. Number of observations used = 31,020.

^b^ No variables were removed from the model.

**FIGURE 5:**
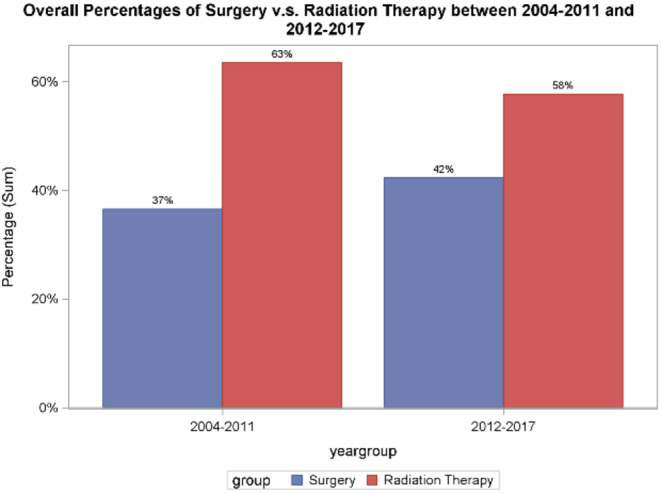
Comparison of percentage of patients undergoing surgery versus radiation therapy (RT), grouped as patients treated from 2004 to 2011 and 2012 to 2017.

## 4 Discussion

This study demonstrated that utilization of RP for GG5 PC has significantly increased in the United States over the past decade, particularly after the USPSTF Grade D recommendation against PSA screening in 2012. As the epidemiologic impact of these recommendations continues to develop, it will be important to investigate evolving practice patterns in the management of this stage disease. Currently there is uncertainty about the best course of management for these patients, as no randomized trials have been completed comparing treatment modalities. The ProtecT trial provided comparison between treatment modalities, however higher risk patients were not well represented [13]. Fortunately there is an ongoing clinical trial, SPCG‐15, that is comparing RP versus definitive radiotherapy for men with high risk prostate cancer [14]. Several retrospective studies have attempted to shed light on this question but have yielded conflicting results. For example, Boorjan et al. reported equivalent 10‐year cancer‐specific survival between RP, RT + ADT, and RT alone in patients with high‐risk disease. However, the risk of all‐cause mortality was greater after RT + ADT compared to RP [15]. Conversely, Kishan et al. found that patients treated with RT + brachytherapy boost + ADT had improved all‐cause mortality compared to patients treated with RT or RP alone [16]. These studies represent only a few of many that have been done to attempt to provide further guidance for these patients [15, 17, 18, 19, 20, 21, 22, 23, 24].

In addition to the aforementioned studies investigating management of high‐risk patients, there have been multiple retrospective contemporary studies that aimed to provide further insight into a comparison of modalities for patients with localized disease. Herlemann et al., using the UCSF‐Cancer of the Prostate Risk Assessment (CAPRA), found that patients undergoing RP for higher‐risk disease had improved survival outcome data compared to RT, brachytherapy (BT), androgen deprivation as primary monotherapy (PADT), and active surveillance/watchful waiting (AS/WW) [25]. Similarly, Knipper et al. evaluated a cohort of patients with Gleason 9–10 disease and found that there was no cancer specific mortality difference (CSM) between RP ± aRT and RT alone. Further, this study indicated that for patients requiring only RP (i.e., No aRT indicated) 10‐year CSM was improved relative to RT alone patients. On the other hand, in a single institution study comparing BT to RP in patients with localized disease, Zhu et al. found that overall survival and cancer specific survival were not different between these 2 groups [26]. These more recent studies highlight the ongoing uncertainty in superior modality for the management of these patients. However, as seen in the Herlemann et al. and Knipper et al. there is retrospective data to support the role of surgical management for high risk patients, which may provide insight into the trend in management strategies identified in this study.

As the present study demonstrates, there has been an increase in the number of patients undergoing prostatectomy for high‐risk disease. This trend may be explained by several different features. As the availability of robotic surgery has increased, perhaps more patients are electing to pursue this treatment option [27]. Additionally, it is also possible that with the increased familiarity with robotic techniques, patients who were previously considered poor surgical candidates are now being offered surgery [28, 29]. Another possible explanation is the aforementioned epidemiologic shift in prostate cancer, in that there are simply more patients being diagnosed with high‐risk disease. This shift may be compounded by the evidence that more patients are being managed with surveillance methods, and thus the treatment focus is being shifted to higher risk patients [30, 31]. As there is retrospective data supporting the use of prostatectomy in high‐risk disease, these patients are being offered surgical management, when previously the operative capacity of surgeons may have been dominated by earlier stage/lower risk patients. Another plausible explanation for patients opting for surgical management is the concern over side effects associated with long courses of ADT that is indicated in high‐risk disease. It is possible and/or likely that this trend in management can be explained by a combination of these factors.

If the trend toward upfront surgery in definitive management for these patients is to continue, it is worthwhile to revisit some of the questions raised by the seminal adjuvant versus salvage radiation studies, including RAVES, GETUG‐AFU 17, and RADICALS. While these trials provided valuable guidance regarding the timing of post‐prostatectomy radiation, patients with high risk and very high‐risk disease were poorly represented [32, 33, 34]. The ARTISTIC meta‐analysis aggregated data from each of the trials to provide summative data on the question of adjuvant versus salvage (i.e., delayed until if/when the PSA recurs) RT. This study found that routine use of adjuvant RT does not improve PSA‐driven EFS in men with localized or locally advanced prostate cancer [35]. While this data is informative, patients with GG5 disease were not well represented in each of the included trials. Specifically, only 9%–17% in the RADICALS, GETUG‐AFU 17, and RAVES trials had Gleason > 8 disease [35]. As such, whether delayed, salvage post‐prostatectomy RT may result in inferior outcomes compared with adjuvant post‐prostatectomy RT remains unclear. With the rise in upfront prostatectomy in these patients, as seen in our analysis, further investigation of post‐prostatectomy timing in these high‐risk patients is needed.

There have been various retrospective studies that have attempted to address this question. Prior to the publication of RAVES, GETUG‐AFU 17, and RADICALS, Hwang et al. performed a multi‐institutional, propensity score matched cohort study of 1566 patients to address the optimal timing for post‐surgery RT in patients with adverse pathologic features. In this study, patients with pT2N0M0/R1 or pT3N0M0/R0‐1 prostate cancer who underwent either adjuvant RT or salvage RT were evaluated. This study found that aRT compared with sRT was associated with higher freedom from biochemical failure (12‐year actuarial rates: 69% vs. 43%) freedom from distant mets (95% vs. 85%), and OS (91% vs. 79%) [36]. This study provide evidence that aRT, rather than sRT, may confer significant benefit to patients with adverse pathologic features after surgery. In a 2018 study, Tilki et al. also sought to address a similar cohort of patients, including those with GG5 localized disease. In this study, “maximal” treatment was described as MaxRT or MaxRP. MaxRP was defined as RP with adjuvant EBRT, ADT or both, while MaxRT was defined as EBRT + brachytherapy + ADT. The results from this study demonstrated that multimodal treatment, whether MaxRT or MaxRP, results in equivalent outcomes in patients with GG5 disease [9]. Ultimately, this study aims to highlight the general trends in primary treatment practices in these high‐risk patients. While results from ongoing trials addressing the comparison of modalities in high‐risk patients are forthcoming, it is worthwhile to also revisit the role of sRT versus aRT, as much of the data driving this decision making was derived from patient populations that are not representative of these current trends. Therefore, further investigation into this question is needed as well.

This study has limitations. First, only patients treated at centers accredited with the Commission on Cancer were included. This database includes approximately 70% of all newly diagnosed cases in the United States and thus captures a significant portion of pertinent data [10]. However, it is important to note that this data is not population‐based and therefore may be missing important cohorts of patients, thus limiting its generalizability [37]. Additionally, it is possible that practice patterns outside commission on cancer centers may diverge from the trends seen in this study. Second, there is no data on rationale for treatment modality. Thus we are unable to evaluate confounding variables such as granular comorbidity (i.e., cardiovascular disease), urinary/erectile function, or prostate anatomy that may bias providers toward RP or RT. Additionally, we did not evaluate the role of robotic surgery, which may explain higher uptake of RP.

## 5 Conclusion

There has been a significant rise in use of RP for men with Gleason grade group 5 prostate cancer in the United States. There has been a particular rise after 2012, when the USPSTF advised against routine PSA screening, which resulted in a decrease in early‐stage cancer and a rise of high‐risk, advanced prostate cancer. While the basis of this treatment shift remains unknown, as more men may pursue upfront RP the role of adjuvant versus early salvage, increased attention to the role of adjuvant versus salvage RT is warranted.

## Author Contributions

**H. Scott McGinnis:** writing – original draft, writing – review and editing. **Taylor Corriher:** writing – original draft, writing – review and editing, methodology. **Sagar A. Patel:** writing – original draft, writing – review and editing, conceptualization, supervision, methodology. **James Janopaul‐Naylor:** writing – review and editing, supervision. **Subir Goyal:** visualization, formal analysis. **Yuan Liu:** visualization, formal analysis. **Zelin Wang:** visualization, formal analysis.

## Ethics Statement

Because this data was de‐identified, the requirement for formal institutional review and need for informed consent were waived, consistent with the policies of Emory University School of Medicine.

## Conflicts of Interest

The authors declare no conflicts of interest.

## Data Availability

The data used in the study is derived from a de‐identified NCDB file and is available upon request to the National Cancer Database. This study adhered to all data policies outlined by *Cancer Medicine*. The American College of Surgeons and the Commission on Cancer have not verified and are not responsible for the analytic or statistical methodology employed, or the conclusions drawn from these data by the investigator. The content is solely the responsibility of the authors and does not necessarily represent the official views of the National Institutes of Health.
